# A diagnostic dilemma: infectious versus noninfectious multifocal choroiditis with panuveitis

**DOI:** 10.1186/1869-5760-3-26

**Published:** 2013-01-28

**Authors:** Sharel Ongchin, C Dirk Keene, Russell Van Gelder, Gurunadh Atma Vemulakonda

**Affiliations:** 1 University of Washington Eye Institute, 325 9th Ave, Box 359608, Seattle, WA, 98104, USA

**Keywords:** Multifocal choroiditis, Sarcoidosis, *Mycobacterium tuberculosis*, Granulomatous inflammation

## Abstract

**Background:**

The objective of this study was to report a diagnostic dilemma in a patient with multifocal choroiditis. This is a case report study.

**Findings:**

A 68-year-old female presented with new onset of floaters in both eyes and diagnosed with bilateral panuveitis. Her visual acuity was 20/200 in both eyes. Slit-lamp examination showed 1+ anterior chamber cells in both eyes. Ophthalmoscopic examination of both eyes showed vitreous cells, optic disc edema, small amounts of subretinal hemorrhage, and punctate choroidal lesions throughout the fundus. Laboratory work-up revealed a positive QuantiFERON-TB Gold result, and the patient was started on antituberculosis medications. However, given the patient’s intolerance to antituberculosis medications and progressive worsening of vision, she underwent a chorioretinal biopsy to assist with determining a definitive diagnosis. Biopsy results showed noncaseating granulomas and were negative for an infectious etiology. The patient was diagnosed with ocular sarcoidosis and started on immunomodulatory therapy for sarcoid-related multifocal choroiditis.

**Conclusions:**

Multifocal chorioretinal lesions of unknown etiology can present as a diagnostic and therapeutic dilemma. Laboratory work-up is useful in determining an etiology; however, more invasive procedures, such as chorioretinal biopsy, may be necessary to guide treatment.

## Findings

### Case presentation

A 68-year-old nurse presented with new onset of floaters in both eyes in August 2009. She noted her symptoms to be painless without eye redness or discharge. She stated that her symptoms progressively worsened with decreasing visual acuity. She was initially seen by an ophthalmologist 2 months after her symptoms started and was diagnosed with bilateral panuveitis. She denied any trauma or injury to the eye, or having any previous ocular history. Her past medical history was significant for insulin-dependent type 2 diabetes mellitus, hypertension, and hypercholesterolemia. She has a history of being purified protein derivative (PPD) positive with a negative chest X-ray, for which she had never received treatment.

**Figure 1:**
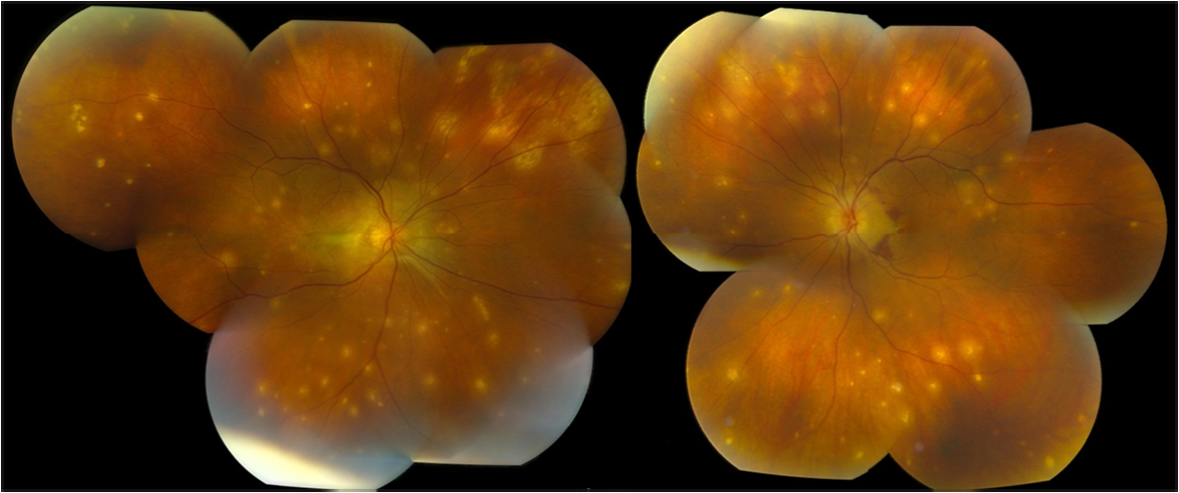
**Dilated fundus examination showing punctate choroidal lesions throughout the fundus in both eyes. **There is also subretinal hemorrhage seen in the left eye.

Her visual acuity was initially 20/200 in the right eye and 20/200 in the left eye. Intraocular pressures were within normal range, and her pupils were equally reactive without the presence of a relative afferent pupillary defect. Slit-lamp examination showed 1+ anterior chamber cells in both eyes. Ophthalmoscopic examination of both eyes showed vitreous cells, optic disc edema, small amounts of subretinal hemorrhage, and punctate choroidal lesions throughout the fundus (Figure 1). 

**Figure 2:**
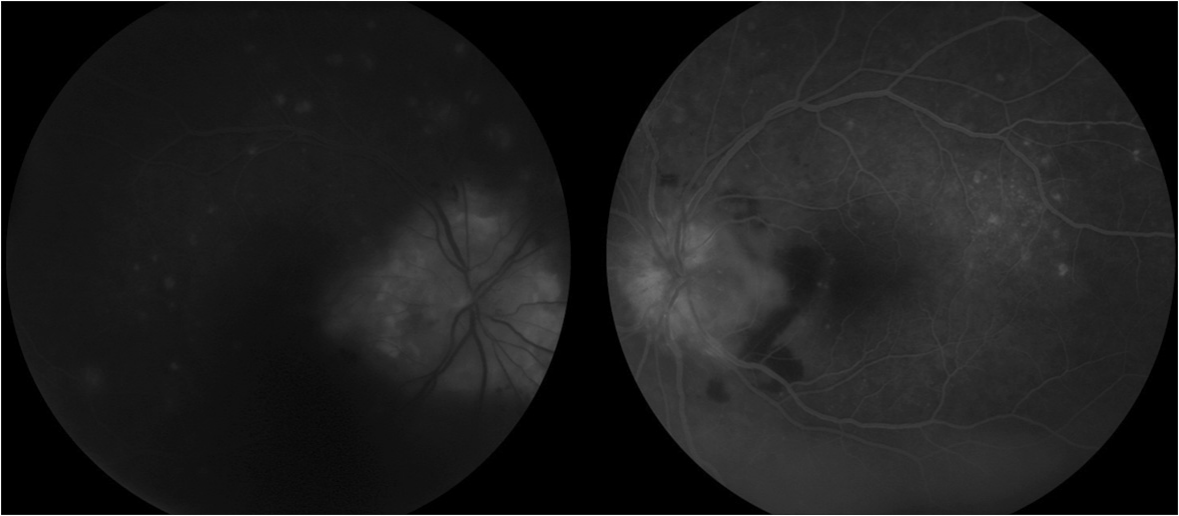
**A fundus fluorescein angiogram. **Early blockage in areas of blood, staining in the location of the choroidal lesions without much leakage, and optic nerve hyperfluorescence bilaterally are shown.

A fluorescein angiogram showed blockage in areas of blood, significant staining in the location of the choroidal lesions without much leakage, and optic nerve hyperfluorescence bilaterally (Figure 2). 

Laboratory investigations revealed a white blood cell count of 11.7 × 10^9^/L. Her erythrocyte sedimentation rate, angiotensin-converting enzyme, liver function test, serum urea and electrolytes, and chest X-ray were normal. *Treponema pallidum* particle agglutination assay was nonreactive, and *Bartonella* titers were negative. She was positive for QuantiFERON-TB Gold, but had a negative chest CT scan and normal brain MRI scan.

After consultation with an infectious disease specialist, she was started on prednisone and antituberculosis medications to cover a possible infection given her positive QuantiFERON-TB Gold test result and prior positive PPD test. The patient developed severe nausea after starting her antituberculosis medications and immediately stopped taking these medications. By this time, her vision had decreased to between 6/200 and 20/500 in the right eye but remained 20/200 in the left eye.

Given the need for immunosuppression along with the patient’s intolerance to antituberculosis medications, a definitive diagnosis for the cause of her choroiditis was urgently needed. As a result, she underwent a pars plana vitrectomy and chorioretinal biopsy of the right eye to confirm whether the etiology of the patient’s multifocal choroiditis was related to a possible extrapulmonary *Mycobacterium tuberculosis* infection.

**Figure 3:**
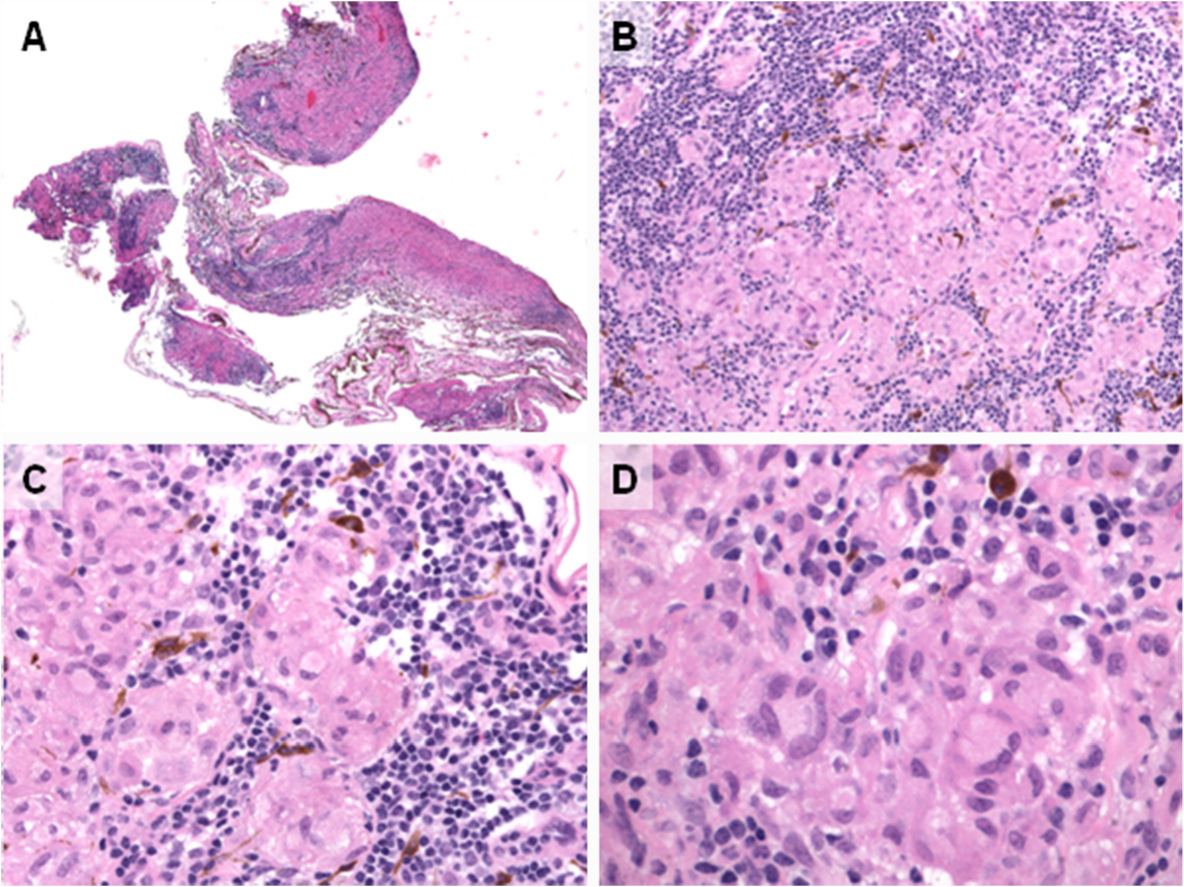
**Choroidal biopsy reveals noncaseating granulomatous inflammation. **H & E-stained slides demonstrate diffuse inflammation expanding fragments of choroid, ×40 (**A**). ×200 magnification reveals dense infiltration of benign-appearing lymphocytes surrounding histiocytic clusters (**B**) which, at higher power (×400), are well circumscribed (**C**) and contain rare multinucleated giant cells, ×600 (**D**).

The biopsy (0.3 × 0.2 × 0.1 cm) was fixed in 4% formaldehyde and processed entirely in paraffin, and multiple sections were cut and stained with hematoxylin and eosin as well as Brown and Brenn tissue gram stain, Ziehl-Neelsen stain, and Fite’s acid-fast stain for mycobacteria, and Grocott’s modified Gomori’s methenamine silver stain for fungal elements. Microscopic examination revealed a small fragment of uveal tissue expanded by inflammation (Figure 3A) comprised of densely infiltrative, benign-appearing lymphocytes surrounding noncaseating histiocyte clusters (Figure 3B) that are well circumscribed (Figure 3C) and contain occasional multinucleated giant cells (Figure 3D) indicative of granulomatous inflammation. No microorganisms were identified. Unstained biopsy sections were submitted for polymerase chain reaction (PCR) testing in which no AFB DNA was detected with 16S rDNA, rpoB, and hsp65 primer sets, no *M. tuberculosis* complex DNA was detected with hsp65-amplified probe, no *Mycobacterium avium* complex DNA was detected with hsp65-amplified probe, and no fungal DNA was detected with 28S rDNA and ITS primer sets. 

Chorioretinal results effectively ruled out an infectious cause for her uveitis. Following this negative biopsy, the patient was diagnosed with ocular sarcoidosis and was started on immunomodulatory therapy for sarcoid-related multifocal choroiditis. She was placed on long-term methotrexate and had resolution of her disc edema, inflammation, and retinal hemorrhages by 1-year follow-up, and stabilization of her chorioretinal lesions. Unfortunately, her visual acuity remained poor given the chronicity of her disease and continued to decline in her right eye secondary to cataract progression.

### Discussion

Patients presenting with multifocal choroiditis and papillitis can be a diagnostic dilemma. Important diagnoses to consider in the differential when managing these patients include tuberculosis, sarcoidosis, histoplasmosis, syphilis, posterior scleritis, intraocular lymphoma, diffuse unilateral subacute neuroretinitis, and Lyme disease. In addition, the various white dot syndromes should also be considered. An initial laboratory work-up should be performed in all patients.

However, even in the setting of positive laboratory results, the diagnosis may still prove to be difficult. Our patient is a nurse and therefore at increased risk for infectious exposure due to her occupational setting. She had a known history of testing positive for purified protein derivative and also had a positive QuantiFERON-TB Gold test result, which would suggest a mycobacterial infectious etiology. Unlike the pulmonary disease, the diagnosis and treatment of ocular tuberculosis is challenging in the absence of clinically apparent pulmonary disease. The diagnosis is complicated by difficulties obtaining ocular tissue, difficulties isolating the organisms from aqueous aspirates, tests that are neither sensitive nor specific, and the variability in clinical presentation [1]. In an analysis by Rosenbaum and Wernick [2] using Bayes’ theorem to analyze the utility of purified protein derivative testing, they calculated that a patient with uveitis and a positive purified protein derivative test result has a 1% likelihood of having tuberculosis. This low probability would suggest low utility in ordering a PPD in routine evaluation of patients with uveitis. Newer testing modalities with interferon-gamma release assays (QuantiFERON-TB Gold or In-Tube and T-SPOT TB) have improved the specificity of the diagnosis as they are more specific markers of *M. tuberculosis* infection and previous exposure and are not influenced by BCG vaccination or exposure to atypical mycobacteria. However, these testing modalities still lack specificity in that they do not distinguish latent tuberculous infection from active disease and may be influenced by immunosuppressive states [3-5].

Culture is still the gold standard for the diagnosis of tuberculosis; however, it may not be readily available and results may take several weeks [3]. Nucleic acid amplification tests, specifically polymerase chain reaction, have emerged as a powerful tool for detection of the mycobacterial genome in clinical and research specimens and indicate high specificity and variable sensitivity, depending on the clinical setting and tested specimens [3,6]. In patients with positive results, quantitative PCR analysis of ocular samples may be useful but still cannot exclude the diagnosis in individuals with negative results because of variable sensitivity and should still take into consideration the entire clinical picture [3,7,8].

Chorioretinal biopsy allowed PCR and special tissue stains which helped guide management in our patient. Given the biopsy results showing noncaseating granulomas in the absence of mycobacterial infection, the patient was diagnosed with and treated for presumed ocular sarcoidosis. A definitive diagnosis of sarcoidosis requires a biopsy showing noncaseating granulomas in the absence of mycobacterial infection. This patient tested positive for QuantiFERON-TB Gold, suggesting mycobacteria as the etiology for the multifocal choroiditis. However, tuberculostatic drugs are associated with significant side effects [9], and therefore, caution should be used when prescribing these medications. Our patient’s intolerance to antituberculosis medications prompted further evaluations including a chorioretinal biopsy which led to the diagnosis of presumed ocular sarcoidosis.

Ocular lesions in sarcoidosis occur in 20% to 30% of patients and are most serious because of the threat of blindness [10]. A variety of clinical patterns of inflammatory disease have been described including isolated anterior uveitis, intermediate uveitis, panuveitis, peripheral retinal vasculitis, isolated or multifocal chorioretinal or retinal pigment epithelial lesions and optic nerve granulomata, or occasionally scleritis. Peripheral multifocal choroiditis occurs mainly in women 55 years of age and is characterized by peripheral punched-out lesions associated with intraocular inflammation. Posterior uveitis may be sight threatening when associated with inflammation of the posterior pole and with cystoid macular edema [11]. Acute anterior uveitis resolves spontaneously or after local therapy with corticosteroids, whereas posterior uveitis oftentimes requires systemic treatment. The key is timely diagnosis in order to promptly treat ocular inflammation.

### Conclusions

Multifocal chorioretinal lesions of unknown etiology can be a diagnostic, and thus therapeutic, dilemma for the physician. Although a laboratory work-up is useful in the diagnosis, it may not always lead to the accurate diagnosis. Although more invasive procedures such as chorioretinal biopsy carry the potential for serious complications, the results may assist in the proper diagnosis and guide management in difficult cases.

### Consent

The research described in this report is in compliance with the Helsinki Declaration. Written informed consent was obtained from the patient for publication of this report and accompanying images.

## Competing interests

The authors declare that they have no competing interests.

## Authors’ contributions

SO wrote the manuscript and accumulated the data. DK was the pathologist who prepared the specimens and tested the tissues for numerous infectious versus inflammatory causes. RVG primarily managed the patient medically and was the primary ophthalmic provider. GAV cared for the patient surgically by performing the chorioretinal biopsy and later medically by initiating immunomodulatory therapy. Both RVG and GAV collaborated on the idea behind the manuscript. All authors read and approved the final manuscript.

## References

[B1] Wroblewski KJ Hidayat AA Neafie RC Rao NA Zapor M Ocular tuberculosis: a clinicopathologic and molecular study Ophthalmology 2011 118 4 772 777 10.1016/j.ophtha.2010.08.011 21055814

[B2] Rosenbaum JT Wernick R The utility of routine screening of patients with uveitis for systemic lupus erythematosus or tuberculosis. A Bayesian analysis Arch Ophthalmol 1990 108 9 1291 1293 10.1001/archopht.1990.01070110107034 2205185

[B3] Dinnes J Deeks J Kunst H Gibson A Cummins E Waugh N Drobniewski F Lalvani A A systemic review of rapid diagnostic tests for the detection of tuberculosis infection Health Technol Assess 2007 11 3 1 196 17266837 10.3310/hta11030

[B4] Albini TA Karakousis PC Rao NA Interferon-gamma release assays in the diagnosis of tuberculous uveitis Am J Ophthalmol 2008 146 4 486 488 10.1016/j.ajo.2008.06.021 18804561

[B5] Pai M Zwerling A Menzies D Systematic review: T-cell-based assays for the diagnosis of latent tuberculosis infection: an update Ann Intern Med 2008 149 3 177 184 18593687 10.7326/0003-4819-149-3-200808050-00241 PMC2951987

[B6] Parashar D Chauhan DS Sharma VD Katoch VM Applications of real-time PCR technology to mycobacterial research Indian J Med Res 2006 124 4 385 398 17159258

[B7] Tan SH Tan BH Goh CL Tan KC Tan MF Ng WC Tan WC Detection of *Mycobacterium tuberculosis* DNA using polymerase chain reaction in cutaneous tuberculosis and tuberculids Int J Dermatol 1999 38 122 127 10.1046/j.1365-4362.1999.00576.x 10192161

[B8] Vasconelos-Santos DV Zierhut M Rao NA Strengths and weaknesses of diagnostic tools to tuberculous uveitis Ocul Immunol Inflamm 2009 17 351 355 10.3109/09273940903168688 19831571 PMC3062469

[B9] Forget EJ Menzies D Adverse reactions to first-line antituberculosis drugs Expert Opin Drug Saf 2006 5 2 231 249 10.1517/14740338.5.2.231 16503745

[B10] James DG Neville E Langley DA Ocular sarcoidosis Trans Ophthalmol Soc UK 1976 96 133 1070842

[B11] Lardenoye CW van der Lelij A de Loos WS Treffers WF Rothova A Peripheral multifocal chorioretinitis: a distinct clinical entity? Ophthalmology 1997 104 1820 1826 9373112 10.1016/s0161-6420(97)30021-9

